# *tert*-Butyl (2*R*,4a*R*,5a*R*,11a*S*,12*R*,12a*R*)-8-[bis­(*tert*-but­oxycarbon­yl)amino]-12-hydroxy-2-meth­oxy-2,10-dioxo-4,4a,5a,6,9,10,11,11a,12,12a-deca­hydro-2*H*-1,3,5-trioxa-6,7,9,11-tetra­aza-2λ^5^-phosphatetra­cene-6-carboxyl­ate methanol monosolvate monohydrate

**DOI:** 10.1107/S160053681202867X

**Published:** 2012-06-30

**Authors:** Graeme J. Gainsford, Keith Clinch, Rachel Dixon, Ashish Tiwari

**Affiliations:** a Carbohydrate Chemistry Group, Industrial Research Limited, PO Box 31-310, Lower Hutt, New Zealand; b Glycosyn Group, Industrial Research Limited, PO Box 31-310, Lower Hutt, New Zealand; c Alexion Pharmaceuticals Inc., 352 Knotter Drive, Cheshire, CT 06410, USA

## Abstract

The title compound, C_26_H_40_N_5_O_13_P·CH_3_OH·H_2_O, crystallizes with one water and one methanol mol­ecule providing important crystal-binding inter­actions. The compound has the unusual feature of having two but­oxy­carbonyl groups bound to one N atom. The conventional attractive hydrogen bonds involving hy­droxy, amine and water donors include bifurcations at both donors and acceptors with novel *R*_1_^2^(6) and *R*_2_^1^(6) motifs. These are supplemented by C—H⋯O inter­actions between adjacent mol­ecules forming chain and *R*_2_^2^(10) ring motifs.

## Related literature

For related structures, see: Low *et al.* (1995 ▶, 1998 ▶, 1999 ▶). For background information, see: Veldman *et al.* (2010 ▶). For ring puckering parameters, see: Cremer & Pople (1975 ▶). For hydrogen-bonding graph-set nomenclature, see: Bernstein *et al.* (1995 ▶).
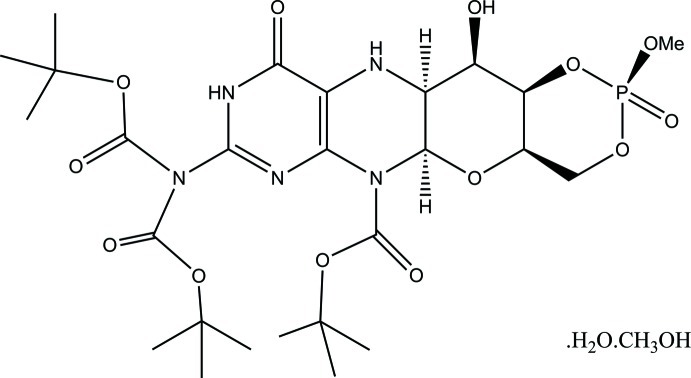


## Experimental

### 

#### Crystal data

C_26_H_40_N_5_O_13_P·CH_4_O·H_2_O
*M* *_r_* = 711.66
Orthorhombic, 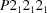

*a* = 10.6435 (4) Å
*b* = 15.9508 (13) Å
*c* = 21.1764 (8) Å
*V* = 3595.2 (3) Å^3^
*Z* = 4
Cu *K*α radiation
μ = 1.31 mm^−1^
*T* = 120 K
0.51 × 0.05 × 0.04 mm

#### Data collection

Oxford Diffraction SuperNova, Dual, Cu at zero, Atlas diffractometer
Absorption correction: multi-scan (*CrysAlis PRO*; Oxford Diffraction, 2007 ▶) *T*_min_ = 0.853, *T*_max_ = 1.000
20603 measured reflections
6796 independent reflections
6210 reflections with *I* > 2σ(*I*)
*R*_int_ = 0.040

#### Refinement

*R*[*F* ^2^ > 2σ(*F* ^2^)] = 0.038
*wR*(*F* ^2^) = 0.096
*S* = 1.04
6796 reflections
452 parameters
2 restraints
H atoms treated by a mixture of independent and constrained refinement
Δρ_max_ = 0.41 e Å^−3^
Δρ_min_ = −0.48 e Å^−3^
Absolute structure: Flack (1983 ▶), 2985 Friedel pairs
Flack parameter: −0.02 (2)

### 

Data collection: *CrysAlis PRO* (Oxford Diffraction, 2007 ▶); cell refinement: *CrysAlis PRO*; data reduction: *CrysAlis PRO*; program(s) used to solve structure: *SHELXS97* (Sheldrick, 2008 ▶); program(s) used to refine structure: *SHELXL97* (Sheldrick, 2008 ▶); molecular graphics: *ORTEP* in *WinGX* (Farrugia, 1999 ▶) and *Mercury* (Macrae *et al.*, 2008 ▶); software used to prepare material for publication: *SHELXL97* and *PLATON* (Spek, 2009 ▶).

## Supplementary Material

Crystal structure: contains datablock(s) global, I. DOI: 10.1107/S160053681202867X/bh2437sup1.cif

Structure factors: contains datablock(s) I. DOI: 10.1107/S160053681202867X/bh2437Isup2.hkl

Supplementary material file. DOI: 10.1107/S160053681202867X/bh2437Isup3.cml

Additional supplementary materials:  crystallographic information; 3D view; checkCIF report

## Figures and Tables

**Table 1:** Hydrogen-bond geometry (Å, °)

*D*—H⋯*A*	*D*—H	H⋯*A*	*D*⋯*A*	*D*—H⋯*A*
N9—H9*N*⋯O14	0.88	1.84	2.718 (3)	179
O12—H12*O*⋯O7^i^	0.84	2.40	3.090 (2)	139
O12—H12*O*⋯O9^i^	0.84	2.32	2.997 (2)	138
O14—H14*A*⋯O15	0.84 (3)	1.97 (3)	2.792 (4)	167 (4)
O14—H14*B*⋯O13^ii^	0.83 (4)	1.97 (4)	2.780 (3)	165 (4)
O15—H15*O*⋯O10	0.84	1.96	2.775 (3)	162
C4—H4*B*⋯O4^iii^	0.99	2.31	3.142 (3)	141
C5*A*—H5*A*⋯O13^iv^	1.00	2.34	3.307 (3)	162
C12*A*—H12*A*⋯O4^iii^	1.00	2.38	3.200 (2)	138
C16—H16*B*⋯O7^v^	0.98	2.44	3.230 (4)	137
C18—H18*B*⋯O10^vi^	0.98	2.38	3.339 (4)	166

Symmetry codes: (i) 
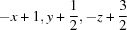
; (ii) 
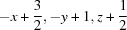
; (iii) 
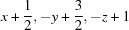
; (iv) 
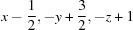
; (v) 
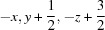
; (vi) 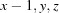.

**Fig. 1.**
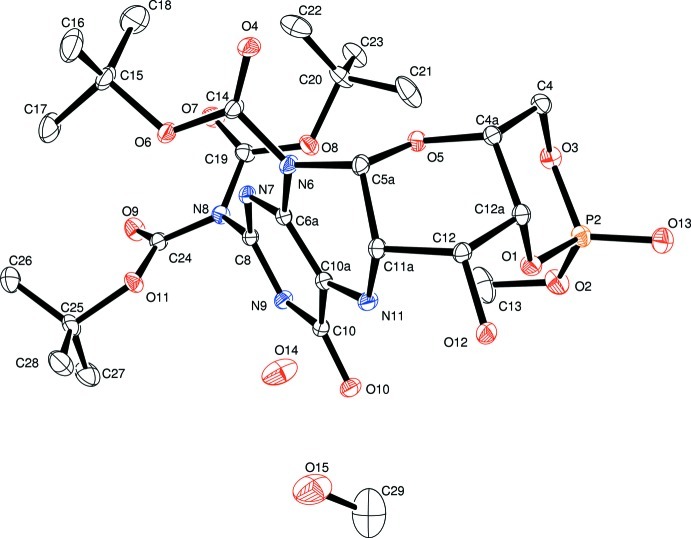
ORTEP (Farrugia, 1999) view of the asymmetric unit atoms with 30% ellipsoid probabilities. H atoms are omitted for clarity.

**Fig. 2.**
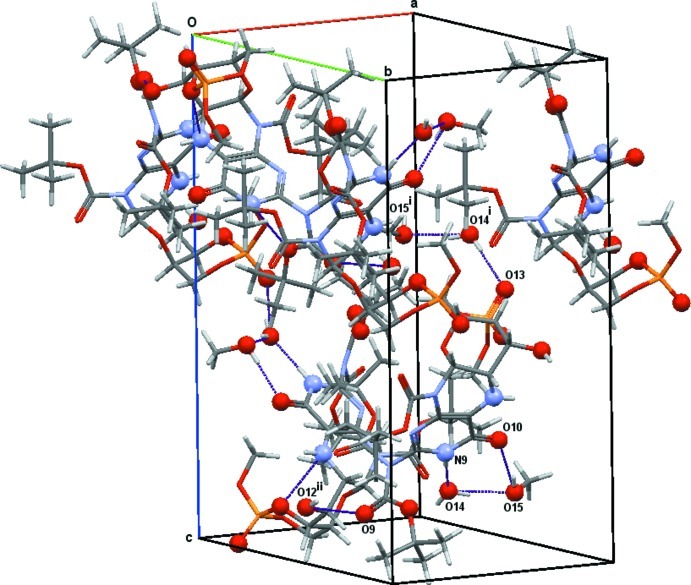
Mercury (Macrae et al., 2008) cell contents view down the ab diagonal; contact atoms are shown as balls. Some intermolecular binding contacts are shown as purple dotted lines. Symmetry: (i) 1.5 - x,1 - y, z - 0.5 (ii) 1 - x, y - 0.5, 1.5 - z.

## supplementary crystallographic information

### Comment

The title compound is an intermediate required for the synthesis of cyclic pyranopterin monophosphate, a molecule whose absence in humans leads to a rare metabolic disorder known as molybdenum cofactor deficiency type A (Veldman *et al.*, 2010).

The title compound crystallizes with one independent C_26_H_40_N_5_O_13_P molecule in the asymmetric unit with one water and one methanol molecule of crystallization (Fig. 1). This and related compounds that were prepared are prone to form merohedrally twinned crystals; this non-twinned structure is the only one successfully solved and refined to date. The absolute configuration was determined using the oxygen anomalous dispersion effect, with the Hooft *y* parameter value calculated as -0.012 (13) (Spek, 2009). This confirms the expected configurations at C4a (*R*), C5a (*R*), C11a (*S*), C12 (*R*) and C12a (*R*).

There are only three closely related structures in the Cambridge Structural Database, namely GODXEC (Low *et al.*, 1999), PUVCIS (Low *et al.*, 1998) and ZENSAM (Low *et al.*, 1995) with none of these involving a fourth fused ring as found here (ring 1, atoms C4a, C12a, O1, P2, O3 & C4). A common feature of all the structures is the buckling of the fused rings progressively away from the near planar ring 4 (C6a, N7, C8, N9, C10 & C10a; see Fig. 1). A comparison of the ring parameters, and their conformational forms (Cremer & Pople, 1975) is given in Table 2 (Ring parameters) with rings 2 & 3 consisting of atoms C5a, C11a, C12, C12a, C4a, O5 and N6, C6a, C10a, N11, C11a, C5a respectively. The similarity of the dihedral angles between the planes for ZENSAM (with the same ring absolute configuration) and PUVCIS (with the inverted configuration) implies that addition of the fourth "capping" ring 1 in the stable chair conformation has not required significant conformational changes to the other rings (see Table 3: Angles between ring planes).

The binding of two BOC groups on nitrogen N8 was slightly unexpected, with the second group expected to bind to the ring nitrogen N9. Indeed, a CSD search (Version 5.33, with Feb. 2012 update) indicates that this occurrence has been reported only 19 times whereas structures with BOC single binding are numbered in the multiple 1000's.

The crystal packing consists of a set of conventional N—H···O(water) and O—H···O(methanol, P, water) hydrogen bonds (see Table 1) which include bifurcations at both donors and acceptors with novel *R*^2^_1_(6) and *R*^1^_2_(6) motifs. These are supplemented by C—H···O interactions between adjacent molecules forming chain and *R*^2^_2_(10) ring motifs (Bernstein *et al.*, 1995).

### Experimental

The details of the synthesis of the title compound will be published elsewhere in due course. The title compound was dissolved in methanol at ambient temperature and diethyl ether added. After 2 h the crystals were filtered off and washed with a little diethyl ether and dried at 15 Torr. The title compound was also crystallized from hot ethanol or hot ethanol and heptane mixtures followed by cooling to ambient temperature but the crystals produced in this way were twinned or were only weakly diffracting.

### Refinement

Seven outlier reflections identified by large delta/sigma ratios (> 4.8) were OMITted from the dataset. All methyl H atoms were constrained to an ideal geometry (C—H = 0.98 Å) with *U*_iso_(H) = 1.5*U*_eq_(C), but were allowed to rotate freely about the adjacent C—C bond. All other C bound H atoms were placed in geometrically idealized positions and constrained to ride on their parent atoms with C—H distances of 1.00 (methine) or 0.99 Å (methylene) and with *U*_iso_(H) = 1.2*U*_eq_(C). hydroxy H atoms on O12 & O15 were constrained to idealized tetrahedral positions with O—H = 0.84 Å, but were allowed to rotate freely about the C—O bond. H atoms on water atom O14 were refined from their difference Fourier map locations, but O—H bond lengths were constrained to 0.84 Å. All hydroxy H atoms were refined with *U*_iso_(H) = 1.5*U*_eq_(O). Finally, N—H bond lengths were fixed to 0.88 Å. A set of 2985 measured Friedel pairs was used to refine the Flack parameter (Flack, 1983).

### Crystal data

**Table:**

C_26_H_40_N_5_O_13_P·CH_4_O·H_2_O	*F*(000) = 1512
*M**_r_* = 711.66	*D*_x_ = 1.315 Mg m^−^^3^
Orthorhombic, *P*2_1_2_1_2_1_	Cu *K*α radiation, λ = 1.54184 Å
Hall symbol: P 2ac 2ab	Cell parameters from 8356 reflections
*a* = 10.6435 (4) Å	θ = 2.8–73.7°
*b* = 15.9508 (13) Å	µ = 1.31 mm^−^^1^
*c* = 21.1764 (8) Å	*T* = 120 K
*V* = 3595.2 (3) Å^3^	Needle, colourless
*Z* = 4	0.51 × 0.05 × 0.04 mm

### Data collection

**Table:**

Oxford Diffraction SuperNova, Dual, Cu at zero, Atlas diffractometer	6796 independent reflections
Radiation source: fine-focus sealed tube	6210 reflections with *I* > 2σ(*I*)
Mirror monochromator	*R*_int_ = 0.040
Detector resolution: 10.6501 pixels mm^-1^	θ_max_ = 70.0°, θ_min_ = 3.5°
ω scans	*h* = −12→12
Absorption correction: multi-scan (*CrysAlis PRO*; Oxford Diffraction, 2007)	*k* = −19→19
*T*_min_ = 0.853, *T*_max_ = 1.000	*l* = −25→25
20603 measured reflections	

### Refinement

**Table:**

Refinement on *F*^2^	Secondary atom site location: difference Fourier map
Least-squares matrix: full	Hydrogen site location: inferred from neighbouring sites
*R*[*F*^2^ > 2σ(*F*^2^)] = 0.038	H atoms treated by a mixture of independent and constrained refinement
*wR*(*F*^2^) = 0.096	*w* = 1/[σ^2^(*F*_o_^2^) + (0.0524*P*)^2^ + 0.416*P*] where *P* = (*F*_o_^2^ + 2*F*_c_^2^)/3
*S* = 1.04	(Δ/σ)_max_ = 0.007
6796 reflections	Δρ_max_ = 0.41 e Å^−^^3^
452 parameters	Δρ_min_ = −0.48 e Å^−^^3^
2 restraints	Absolute structure: Flack (1983), 2985 Friedel pairs
0 constraints	Flack parameter: −0.02 (2)
Primary atom site location: structure-invariant direct methods	

### Fractional atomic coordinates and isotropic or equivalent isotropic displacement parameters (Å^2^)

**Table:**

	*x*	*y*	*z*	*U*_iso_*/*U*_eq_	
P2	0.80446 (5)	0.58835 (3)	0.54460 (3)	0.02445 (12)	
O1	0.78734 (14)	0.68097 (9)	0.57026 (7)	0.0244 (3)	
O2	0.84799 (17)	0.53670 (10)	0.60281 (8)	0.0340 (4)	
O3	0.66670 (15)	0.55698 (10)	0.53112 (7)	0.0270 (3)	
O4	0.22697 (14)	0.79511 (11)	0.61522 (7)	0.0297 (4)	
O5	0.50882 (13)	0.69304 (9)	0.57972 (7)	0.0219 (3)	
O6	0.24587 (13)	0.79463 (10)	0.72208 (7)	0.0246 (3)	
O7	0.21893 (16)	0.46434 (11)	0.79895 (8)	0.0340 (4)	
O8	0.37798 (15)	0.48589 (10)	0.72995 (7)	0.0285 (3)	
O9	0.29577 (16)	0.52087 (10)	0.91740 (7)	0.0307 (3)	
O10	0.77295 (13)	0.67374 (10)	0.77704 (7)	0.0256 (3)	
O11	0.38713 (14)	0.64782 (10)	0.89850 (7)	0.0253 (3)	
O12	0.81909 (13)	0.84935 (9)	0.58518 (7)	0.0239 (3)	
H12O	0.8140	0.8978	0.6006	0.036*	
O13	0.89193 (15)	0.57970 (11)	0.49161 (8)	0.0313 (3)	
N6	0.41153 (16)	0.76019 (11)	0.66317 (8)	0.0215 (3)	
N7	0.39592 (15)	0.66294 (11)	0.74668 (8)	0.0200 (3)	
N8	0.38213 (16)	0.55516 (11)	0.82093 (8)	0.0215 (3)	
N9	0.57866 (16)	0.61801 (11)	0.79852 (8)	0.0215 (3)	
H9N	0.6101	0.5847	0.8276	0.026*	
N11	0.66929 (16)	0.76924 (11)	0.67840 (8)	0.0227 (4)	
H11N	0.7509	0.7731	0.6845	0.027*	
C4	0.5850 (2)	0.61209 (14)	0.49446 (10)	0.0248 (4)	
H4A	0.4995	0.5879	0.4930	0.030*	
H4B	0.6168	0.6155	0.4506	0.030*	
C4A	0.57849 (19)	0.69937 (14)	0.52219 (9)	0.0220 (4)	
H4AA	0.5311	0.7362	0.4923	0.026*	
C5A	0.48696 (18)	0.77318 (13)	0.60754 (9)	0.0208 (4)	
H5A	0.4409	0.8099	0.5771	0.025*	
C6A	0.46909 (19)	0.71540 (13)	0.71201 (9)	0.0200 (4)	
C8	0.45310 (18)	0.61650 (13)	0.78714 (9)	0.0194 (4)	
C10	0.65855 (18)	0.67069 (13)	0.76551 (9)	0.0201 (4)	
C10A	0.59833 (19)	0.72028 (13)	0.71732 (9)	0.0200 (4)	
C11A	0.61092 (18)	0.81506 (13)	0.62689 (9)	0.0199 (4)	
H11A	0.5922	0.8733	0.6417	0.024*	
C12	0.69743 (19)	0.82063 (12)	0.56888 (9)	0.0192 (4)	
H12	0.6600	0.8625	0.5392	0.023*	
C12A	0.70849 (19)	0.73790 (13)	0.53355 (9)	0.0212 (4)	
H12A	0.7496	0.7484	0.4918	0.025*	
C13	0.7751 (4)	0.5374 (2)	0.66060 (13)	0.0540 (8)	
H13A	0.7075	0.5788	0.6570	0.081*	
H13B	0.8298	0.5521	0.6962	0.081*	
H13C	0.7389	0.4817	0.6677	0.081*	
C14	0.28548 (18)	0.78421 (13)	0.66324 (10)	0.0219 (4)	
C15	0.1099 (2)	0.79637 (19)	0.73616 (11)	0.0344 (5)	
C16	0.0540 (3)	0.8768 (2)	0.71126 (14)	0.0550 (9)	
H16A	0.1050	0.9244	0.7253	0.082*	
H16B	−0.0319	0.8830	0.7273	0.082*	
H16C	0.0524	0.8751	0.6650	0.082*	
C17	0.1096 (3)	0.7949 (2)	0.80794 (12)	0.0453 (7)	
H17A	0.1536	0.7447	0.8228	0.068*	
H17B	0.0227	0.7941	0.8233	0.068*	
H17C	0.1523	0.8450	0.8240	0.068*	
C18	0.0484 (3)	0.7190 (2)	0.70969 (16)	0.0552 (8)	
H18A	0.0465	0.7226	0.6635	0.083*	
H18B	−0.0376	0.7148	0.7258	0.083*	
H18C	0.0963	0.6694	0.7225	0.083*	
C19	0.3147 (2)	0.49719 (13)	0.78327 (10)	0.0244 (4)	
C20	0.3148 (3)	0.45903 (14)	0.67033 (11)	0.0330 (5)	
C21	0.4166 (3)	0.4769 (2)	0.62155 (12)	0.0517 (8)	
H21A	0.4359	0.5370	0.6215	0.078*	
H21B	0.3868	0.4602	0.5796	0.078*	
H21C	0.4925	0.4451	0.6322	0.078*	
C22	0.1987 (3)	0.51106 (18)	0.65935 (13)	0.0475 (7)	
H22A	0.1349	0.4966	0.6909	0.071*	
H22B	0.1657	0.4997	0.6170	0.071*	
H22C	0.2199	0.5707	0.6630	0.071*	
C23	0.2869 (3)	0.36592 (15)	0.67444 (12)	0.0382 (6)	
H23A	0.3648	0.3352	0.6828	0.057*	
H23B	0.2506	0.3467	0.6344	0.057*	
H23C	0.2270	0.3557	0.7088	0.057*	
C24	0.34876 (19)	0.57038 (13)	0.88421 (10)	0.0227 (4)	
C25	0.3793 (2)	0.68166 (16)	0.96382 (10)	0.0301 (5)	
C26	0.2439 (3)	0.6844 (2)	0.98517 (12)	0.0409 (6)	
H26A	0.1918	0.7084	0.9515	0.061*	
H26B	0.2371	0.7193	1.0231	0.061*	
H26C	0.2151	0.6275	0.9947	0.061*	
C27	0.4631 (3)	0.6298 (2)	1.00673 (12)	0.0437 (6)	
H27A	0.4300	0.5726	1.0096	0.065*	
H27B	0.4650	0.6551	1.0489	0.065*	
H27C	0.5484	0.6283	0.9894	0.065*	
C28	0.4294 (3)	0.77006 (17)	0.95447 (13)	0.0410 (6)	
H28A	0.5139	0.7674	0.9362	0.061*	
H28B	0.4329	0.7989	0.9953	0.061*	
H28C	0.3736	0.8009	0.9259	0.061*	
O14	0.6796 (2)	0.51634 (16)	0.88814 (11)	0.0581 (6)	
H14A	0.742 (3)	0.547 (2)	0.895 (2)	0.087*	
H14B	0.664 (5)	0.480 (2)	0.9153 (18)	0.087*	
O15	0.8836 (3)	0.6263 (2)	0.89018 (13)	0.0791 (9)	
H15O	0.8575	0.6507	0.8576	0.119*	
C29	0.9845 (6)	0.5826 (3)	0.8763 (3)	0.0947 (17)	
H29A	1.0130	0.5523	0.9140	0.142*	
H29B	1.0511	0.6205	0.8619	0.142*	
H29C	0.9647	0.5424	0.8428	0.142*	

### Atomic displacement parameters (Å^2^)

**Table:**

	*U*^11^	*U*^22^	*U*^33^	*U*^12^	*U*^13^	*U*^23^
P2	0.0273 (3)	0.0234 (2)	0.0227 (3)	0.0025 (2)	0.0006 (2)	−0.0004 (2)
O1	0.0240 (7)	0.0249 (7)	0.0243 (7)	0.0034 (6)	−0.0025 (5)	−0.0016 (6)
O2	0.0429 (9)	0.0303 (9)	0.0288 (8)	0.0091 (7)	−0.0041 (7)	0.0011 (7)
O3	0.0305 (8)	0.0234 (7)	0.0271 (8)	−0.0025 (6)	0.0028 (6)	0.0003 (6)
O4	0.0209 (7)	0.0494 (10)	0.0187 (7)	0.0034 (6)	−0.0031 (6)	0.0043 (7)
O5	0.0214 (7)	0.0267 (7)	0.0177 (7)	−0.0032 (6)	0.0028 (5)	0.0018 (6)
O6	0.0173 (7)	0.0369 (9)	0.0195 (7)	0.0043 (6)	0.0020 (5)	0.0023 (6)
O7	0.0318 (9)	0.0413 (9)	0.0288 (8)	−0.0155 (7)	0.0027 (6)	−0.0010 (7)
O8	0.0324 (8)	0.0334 (9)	0.0197 (7)	−0.0061 (7)	0.0017 (6)	−0.0031 (6)
O9	0.0401 (9)	0.0298 (8)	0.0222 (7)	−0.0066 (7)	0.0066 (7)	0.0043 (6)
O10	0.0180 (7)	0.0355 (8)	0.0233 (7)	−0.0013 (6)	−0.0010 (5)	0.0048 (6)
O11	0.0312 (8)	0.0300 (8)	0.0147 (7)	−0.0061 (6)	0.0038 (6)	−0.0010 (6)
O12	0.0206 (7)	0.0257 (7)	0.0252 (7)	−0.0036 (6)	0.0039 (6)	−0.0030 (6)
O13	0.0320 (8)	0.0292 (8)	0.0326 (8)	0.0029 (7)	0.0046 (7)	−0.0019 (7)
N6	0.0163 (8)	0.0308 (9)	0.0175 (8)	0.0020 (7)	0.0015 (6)	0.0044 (7)
N7	0.0171 (8)	0.0264 (9)	0.0164 (8)	−0.0012 (7)	0.0028 (6)	0.0012 (7)
N8	0.0226 (8)	0.0232 (8)	0.0186 (8)	−0.0042 (7)	0.0025 (6)	0.0028 (7)
N9	0.0187 (8)	0.0273 (9)	0.0184 (8)	0.0005 (7)	0.0006 (6)	0.0048 (7)
N11	0.0180 (8)	0.0331 (9)	0.0171 (8)	−0.0026 (7)	0.0006 (6)	0.0042 (7)
C4	0.0258 (10)	0.0300 (11)	0.0185 (10)	−0.0035 (8)	0.0003 (8)	−0.0001 (8)
C4A	0.0206 (9)	0.0302 (11)	0.0153 (9)	−0.0009 (8)	0.0007 (7)	0.0017 (8)
C5A	0.0196 (9)	0.0259 (10)	0.0169 (9)	0.0015 (8)	0.0031 (7)	0.0025 (8)
C6A	0.0180 (9)	0.0271 (10)	0.0148 (9)	−0.0004 (8)	0.0016 (7)	−0.0019 (8)
C8	0.0176 (9)	0.0252 (10)	0.0154 (9)	−0.0018 (7)	0.0023 (7)	0.0005 (8)
C10	0.0167 (9)	0.0262 (10)	0.0175 (9)	0.0011 (7)	0.0010 (7)	−0.0008 (8)
C10A	0.0187 (9)	0.0257 (10)	0.0157 (9)	−0.0011 (8)	0.0011 (7)	−0.0009 (8)
C11A	0.0196 (9)	0.0234 (10)	0.0165 (9)	−0.0015 (8)	0.0033 (7)	0.0012 (7)
C12	0.0183 (9)	0.0214 (9)	0.0178 (9)	−0.0019 (8)	0.0023 (7)	0.0027 (7)
C12A	0.0218 (9)	0.0254 (10)	0.0165 (9)	−0.0019 (8)	0.0035 (7)	0.0026 (7)
C13	0.085 (2)	0.0464 (16)	0.0303 (14)	0.0231 (16)	0.0068 (14)	0.0051 (12)
C14	0.0151 (9)	0.0291 (10)	0.0215 (10)	0.0003 (8)	0.0025 (7)	0.0038 (8)
C15	0.0171 (10)	0.0594 (16)	0.0267 (11)	0.0071 (10)	0.0072 (8)	0.0037 (11)
C16	0.0445 (16)	0.085 (2)	0.0358 (15)	0.0371 (16)	0.0060 (12)	0.0046 (15)
C17	0.0351 (14)	0.0730 (19)	0.0277 (12)	0.0121 (13)	0.0130 (10)	0.0063 (13)
C18	0.0316 (14)	0.083 (2)	0.0508 (17)	−0.0212 (14)	0.0124 (12)	−0.0025 (16)
C19	0.0277 (10)	0.0263 (10)	0.0193 (10)	−0.0035 (9)	−0.0012 (8)	0.0038 (8)
C20	0.0506 (14)	0.0306 (11)	0.0178 (10)	−0.0100 (11)	−0.0032 (10)	−0.0012 (9)
C21	0.082 (2)	0.0490 (16)	0.0245 (13)	−0.0280 (16)	0.0135 (13)	−0.0056 (11)
C22	0.0617 (17)	0.0460 (15)	0.0350 (14)	0.0003 (14)	−0.0203 (13)	0.0022 (11)
C23	0.0573 (16)	0.0320 (12)	0.0255 (11)	−0.0112 (11)	0.0005 (11)	−0.0010 (10)
C24	0.0211 (9)	0.0281 (11)	0.0190 (10)	−0.0004 (8)	0.0007 (7)	0.0019 (8)
C25	0.0361 (12)	0.0379 (13)	0.0162 (10)	−0.0013 (10)	0.0030 (8)	−0.0055 (9)
C26	0.0391 (14)	0.0532 (17)	0.0304 (13)	−0.0011 (11)	0.0105 (10)	−0.0082 (11)
C27	0.0549 (16)	0.0520 (16)	0.0240 (12)	0.0032 (13)	−0.0065 (11)	−0.0012 (11)
C28	0.0504 (15)	0.0407 (14)	0.0318 (13)	−0.0084 (11)	0.0015 (11)	−0.0098 (11)
O14	0.0417 (11)	0.0749 (16)	0.0576 (13)	−0.0065 (10)	−0.0128 (10)	0.0422 (12)
O15	0.0619 (15)	0.116 (2)	0.0595 (15)	−0.0051 (16)	−0.0190 (13)	0.0278 (16)
C29	0.131 (4)	0.049 (2)	0.104 (4)	0.011 (3)	0.042 (3)	0.019 (2)

### Geometric parameters (Å, º)

**Table:**

P2—O13	1.4647 (17)	C12A—H12A	1.0000
P2—O2	1.5533 (17)	C13—H13A	0.9800
P2—O3	1.5755 (16)	C13—H13B	0.9800
P2—O1	1.5845 (16)	C13—H13C	0.9800
O1—C12A	1.461 (2)	C15—C18	1.505 (4)
O2—C13	1.449 (3)	C15—C16	1.509 (4)
O3—C4	1.460 (3)	C15—C17	1.520 (3)
O4—C14	1.205 (3)	C16—H16A	0.9800
O5—C5A	1.427 (3)	C16—H16B	0.9800
O5—C4A	1.430 (2)	C16—H16C	0.9800
O6—C14	1.326 (3)	C17—H17A	0.9800
O6—C15	1.478 (3)	C17—H17B	0.9800
O7—C19	1.193 (3)	C17—H17C	0.9800
O8—C19	1.327 (3)	C18—H18A	0.9800
O8—C20	1.493 (3)	C18—H18B	0.9800
O9—C24	1.198 (3)	C18—H18C	0.9800
O10—C10	1.243 (3)	C20—C22	1.507 (4)
O11—C24	1.336 (3)	C20—C23	1.517 (3)
O11—C25	1.487 (3)	C20—C21	1.523 (4)
O12—C12	1.416 (3)	C21—H21A	0.9800
O12—H12O	0.8400	C21—H21B	0.9800
N6—C14	1.395 (3)	C21—H21C	0.9800
N6—C6A	1.398 (3)	C22—H22A	0.9800
N6—C5A	1.441 (3)	C22—H22B	0.9800
N7—C8	1.286 (3)	C22—H22C	0.9800
N7—C6A	1.359 (3)	C23—H23A	0.9800
N8—C24	1.407 (3)	C23—H23B	0.9800
N8—C19	1.416 (3)	C23—H23C	0.9800
N8—C8	1.428 (3)	C25—C26	1.511 (3)
N9—C8	1.358 (3)	C25—C27	1.518 (4)
N9—C10	1.385 (3)	C25—C28	1.520 (4)
N9—H9N	0.8800	C26—H26A	0.9800
N11—C10A	1.364 (3)	C26—H26B	0.9800
N11—C11A	1.453 (3)	C26—H26C	0.9800
N11—H11N	0.8800	C27—H27A	0.9800
C4—C4A	1.513 (3)	C27—H27B	0.9800
C4—H4A	0.9900	C27—H27C	0.9800
C4—H4B	0.9900	C28—H28A	0.9800
C4A—C12A	1.533 (3)	C28—H28B	0.9800
C4A—H4AA	1.0000	C28—H28C	0.9800
C5A—C11A	1.535 (3)	O14—H14A	0.83 (2)
C5A—H5A	1.0000	O14—H14B	0.829 (19)
C6A—C10A	1.382 (3)	O15—C29	1.313 (6)
C10—C10A	1.442 (3)	O15—H15O	0.8400
C11A—C12	1.538 (2)	C29—H29A	0.9800
C11A—H11A	1.0000	C29—H29B	0.9800
C12—C12A	1.522 (3)	C29—H29C	0.9800
C12—H12	1.0000		
			
O13—P2—O2	111.63 (10)	O6—C15—C16	109.4 (2)
O13—P2—O3	115.00 (9)	C18—C15—C16	113.2 (3)
O2—P2—O3	104.65 (9)	O6—C15—C17	101.75 (18)
O13—P2—O1	115.07 (9)	C18—C15—C17	111.1 (3)
O2—P2—O1	104.88 (9)	C16—C15—C17	111.2 (2)
O3—P2—O1	104.55 (9)	C15—C16—H16A	109.5
C12A—O1—P2	117.60 (13)	C15—C16—H16B	109.5
C13—O2—P2	120.45 (17)	H16A—C16—H16B	109.5
C4—O3—P2	117.34 (13)	C15—C16—H16C	109.5
C5A—O5—C4A	111.91 (15)	H16A—C16—H16C	109.5
C14—O6—C15	120.22 (16)	H16B—C16—H16C	109.5
C19—O8—C20	122.02 (18)	C15—C17—H17A	109.5
C24—O11—C25	121.94 (17)	C15—C17—H17B	109.5
C12—O12—H12O	109.5	H17A—C17—H17B	109.5
C14—N6—C6A	124.11 (17)	C15—C17—H17C	109.5
C14—N6—C5A	119.81 (17)	H17A—C17—H17C	109.5
C6A—N6—C5A	115.74 (16)	H17B—C17—H17C	109.5
C8—N7—C6A	116.33 (17)	C15—C18—H18A	109.5
C24—N8—C19	121.39 (17)	C15—C18—H18B	109.5
C24—N8—C8	119.50 (17)	H18A—C18—H18B	109.5
C19—N8—C8	115.65 (16)	C15—C18—H18C	109.5
C8—N9—C10	121.68 (18)	H18A—C18—H18C	109.5
C8—N9—H9N	119.2	H18B—C18—H18C	109.5
C10—N9—H9N	119.2	O7—C19—O8	127.6 (2)
C10A—N11—C11A	120.34 (16)	O7—C19—N8	124.2 (2)
C10A—N11—H11N	119.8	O8—C19—N8	108.11 (17)
C11A—N11—H11N	119.8	O8—C20—C22	110.0 (2)
O3—C4—C4A	112.04 (16)	O8—C20—C23	108.70 (19)
O3—C4—H4A	109.2	C22—C20—C23	112.8 (2)
C4A—C4—H4A	109.2	O8—C20—C21	101.5 (2)
O3—C4—H4B	109.2	C22—C20—C21	112.0 (2)
C4A—C4—H4B	109.2	C23—C20—C21	111.2 (2)
H4A—C4—H4B	107.9	C20—C21—H21A	109.5
O5—C4A—C4	106.84 (17)	C20—C21—H21B	109.5
O5—C4A—C12A	111.27 (16)	H21A—C21—H21B	109.5
C4—C4A—C12A	112.84 (17)	C20—C21—H21C	109.5
O5—C4A—H4AA	108.6	H21A—C21—H21C	109.5
C4—C4A—H4AA	108.6	H21B—C21—H21C	109.5
C12A—C4A—H4AA	108.6	C20—C22—H22A	109.5
O5—C5A—N6	107.44 (16)	C20—C22—H22B	109.5
O5—C5A—C11A	111.11 (16)	H22A—C22—H22B	109.5
N6—C5A—C11A	108.87 (16)	C20—C22—H22C	109.5
O5—C5A—H5A	109.8	H22A—C22—H22C	109.5
N6—C5A—H5A	109.8	H22B—C22—H22C	109.5
C11A—C5A—H5A	109.8	C20—C23—H23A	109.5
N7—C6A—C10A	124.14 (19)	C20—C23—H23B	109.5
N7—C6A—N6	117.59 (18)	H23A—C23—H23B	109.5
C10A—C6A—N6	117.86 (18)	C20—C23—H23C	109.5
N7—C8—N9	125.02 (19)	H23A—C23—H23C	109.5
N7—C8—N8	118.57 (18)	H23B—C23—H23C	109.5
N9—C8—N8	116.33 (18)	O9—C24—O11	128.4 (2)
O10—C10—N9	121.75 (19)	O9—C24—N8	124.3 (2)
O10—C10—C10A	123.58 (19)	O11—C24—N8	107.33 (17)
N9—C10—C10A	114.67 (17)	O11—C25—C26	110.04 (19)
N11—C10A—C6A	122.29 (19)	O11—C25—C27	109.03 (19)
N11—C10A—C10	119.73 (18)	C26—C25—C27	113.4 (2)
C6A—C10A—C10	117.98 (19)	O11—C25—C28	101.29 (18)
N11—C11A—C5A	110.44 (16)	C26—C25—C28	110.3 (2)
N11—C11A—C12	111.89 (16)	C27—C25—C28	112.1 (2)
C5A—C11A—C12	109.07 (16)	C25—C26—H26A	109.5
N11—C11A—H11A	108.5	C25—C26—H26B	109.5
C5A—C11A—H11A	108.5	H26A—C26—H26B	109.5
C12—C11A—H11A	108.5	C25—C26—H26C	109.5
O12—C12—C12A	109.25 (16)	H26A—C26—H26C	109.5
O12—C12—C11A	111.80 (16)	H26B—C26—H26C	109.5
C12A—C12—C11A	112.89 (16)	C25—C27—H27A	109.5
O12—C12—H12	107.6	C25—C27—H27B	109.5
C12A—C12—H12	107.6	H27A—C27—H27B	109.5
C11A—C12—H12	107.6	C25—C27—H27C	109.5
O1—C12A—C12	108.78 (15)	H27A—C27—H27C	109.5
O1—C12A—C4A	110.67 (16)	H27B—C27—H27C	109.5
C12—C12A—C4A	110.79 (17)	C25—C28—H28A	109.5
O1—C12A—H12A	108.9	C25—C28—H28B	109.5
C12—C12A—H12A	108.9	H28A—C28—H28B	109.5
C4A—C12A—H12A	108.9	C25—C28—H28C	109.5
O2—C13—H13A	109.5	H28A—C28—H28C	109.5
O2—C13—H13B	109.5	H28B—C28—H28C	109.5
H13A—C13—H13B	109.5	H14A—O14—H14B	117 (5)
O2—C13—H13C	109.5	C29—O15—H15O	109.5
H13A—C13—H13C	109.5	O15—C29—H29A	109.5
H13B—C13—H13C	109.5	O15—C29—H29B	109.5
O4—C14—O6	127.63 (18)	H29A—C29—H29B	109.5
O4—C14—N6	122.40 (19)	O15—C29—H29C	109.5
O6—C14—N6	109.94 (17)	H29A—C29—H29C	109.5
O6—C15—C18	109.6 (2)	H29B—C29—H29C	109.5
			
O13—P2—O1—C12A	77.02 (16)	C10A—N11—C11A—C12	−145.40 (18)
O2—P2—O1—C12A	−159.94 (14)	O5—C5A—C11A—N11	−67.4 (2)
O3—P2—O1—C12A	−50.11 (15)	N6—C5A—C11A—N11	50.7 (2)
O13—P2—O2—C13	179.4 (2)	O5—C5A—C11A—C12	56.0 (2)
O3—P2—O2—C13	−55.6 (2)	N6—C5A—C11A—C12	174.10 (16)
O1—P2—O2—C13	54.1 (2)	N11—C11A—C12—O12	−50.3 (2)
O13—P2—O3—C4	−78.07 (16)	C5A—C11A—C12—O12	−172.83 (16)
O2—P2—O3—C4	159.09 (14)	N11—C11A—C12—C12A	73.3 (2)
O1—P2—O3—C4	49.09 (16)	C5A—C11A—C12—C12A	−49.2 (2)
P2—O3—C4—C4A	−53.3 (2)	P2—O1—C12A—C12	175.96 (12)
C5A—O5—C4A—C4	−174.89 (16)	P2—O1—C12A—C4A	54.02 (19)
C5A—O5—C4A—C12A	61.5 (2)	O12—C12—C12A—O1	51.19 (19)
O3—C4—C4A—O5	−70.5 (2)	C11A—C12—C12A—O1	−73.9 (2)
O3—C4—C4A—C12A	52.1 (2)	O12—C12—C12A—C4A	173.05 (15)
C4A—O5—C5A—N6	177.32 (15)	C11A—C12—C12A—C4A	48.0 (2)
C4A—O5—C5A—C11A	−63.7 (2)	O5—C4A—C12A—O1	67.9 (2)
C14—N6—C5A—O5	−107.9 (2)	C4—C4A—C12A—O1	−52.1 (2)
C6A—N6—C5A—O5	65.7 (2)	O5—C4A—C12A—C12	−52.8 (2)
C14—N6—C5A—C11A	131.70 (19)	C4—C4A—C12A—C12	−172.90 (16)
C6A—N6—C5A—C11A	−54.7 (2)	C15—O6—C14—O4	19.3 (4)
C8—N7—C6A—C10A	0.9 (3)	C15—O6—C14—N6	−162.6 (2)
C8—N7—C6A—N6	173.44 (18)	C6A—N6—C14—O4	−153.2 (2)
C14—N6—C6A—N7	28.9 (3)	C5A—N6—C14—O4	19.8 (3)
C5A—N6—C6A—N7	−144.36 (18)	C6A—N6—C14—O6	28.6 (3)
C14—N6—C6A—C10A	−158.1 (2)	C5A—N6—C14—O6	−158.35 (18)
C5A—N6—C6A—C10A	28.6 (3)	C14—O6—C15—C18	53.6 (3)
C6A—N7—C8—N9	1.9 (3)	C14—O6—C15—C16	−71.1 (3)
C6A—N7—C8—N8	−174.69 (17)	C14—O6—C15—C17	171.2 (2)
C10—N9—C8—N7	−1.1 (3)	C20—O8—C19—O7	26.9 (3)
C10—N9—C8—N8	175.53 (18)	C20—O8—C19—N8	−154.39 (18)
C24—N8—C8—N7	−103.6 (2)	C24—N8—C19—O7	8.5 (3)
C19—N8—C8—N7	55.7 (3)	C8—N8—C19—O7	−150.5 (2)
C24—N8—C8—N9	79.5 (2)	C24—N8—C19—O8	−170.32 (18)
C19—N8—C8—N9	−121.1 (2)	C8—N8—C19—O8	30.8 (2)
C8—N9—C10—O10	178.0 (2)	C19—O8—C20—C22	47.2 (3)
C8—N9—C10—C10A	−2.3 (3)	C19—O8—C20—C23	−76.6 (3)
C11A—N11—C10A—C6A	−3.5 (3)	C19—O8—C20—C21	166.0 (2)
C11A—N11—C10A—C10	175.77 (18)	C25—O11—C24—O9	6.4 (3)
N7—C6A—C10A—N11	175.01 (19)	C25—O11—C24—N8	−173.45 (17)
N6—C6A—C10A—N11	2.5 (3)	C19—N8—C24—O9	26.3 (3)
N7—C6A—C10A—C10	−4.3 (3)	C8—N8—C24—O9	−175.6 (2)
N6—C6A—C10A—C10	−176.77 (18)	C19—N8—C24—O11	−153.78 (18)
O10—C10—C10A—N11	5.0 (3)	C8—N8—C24—O11	4.4 (2)
N9—C10—C10A—N11	−174.64 (18)	C24—O11—C25—C26	−61.7 (3)
O10—C10—C10A—C6A	−175.7 (2)	C24—O11—C25—C27	63.3 (3)
N9—C10—C10A—C6A	4.7 (3)	C24—O11—C25—C28	−178.3 (2)
C10A—N11—C11A—C5A	−23.7 (3)		

### Hydrogen-bond geometry (Å, º)

**Table:**

*D*—H···*A*	*D*—H	H···*A*	*D*···*A*	*D*—H···*A*
N9—H9*N*···O14	0.88	1.84	2.718 (3)	179
O12—H12*O*···O7^i^	0.84	2.40	3.090 (2)	139
O12—H12*O*···O9^i^	0.84	2.32	2.997 (2)	138
O14—H14*A*···O15	0.84 (3)	1.97 (3)	2.792 (4)	167 (4)
O14—H14*B*···O13^ii^	0.83 (4)	1.97 (4)	2.780 (3)	165 (4)
O15—H15*O*···O10	0.84	1.96	2.775 (3)	162
C4—H4*B*···O4^iii^	0.99	2.31	3.142 (3)	141
C5*A*—H5*A*···O13^iv^	1.00	2.34	3.307 (3)	162
C12*A*—H12*A*···O4^iii^	1.00	2.38	3.200 (2)	138
C16—H16*B*···O7^v^	0.98	2.44	3.230 (4)	137
C18—H18*B*···O10^vi^	0.98	2.38	3.339 (4)	166

Symmetry codes: (i) −*x*+1, *y*+1/2, −*z*+3/2; (ii) −*x*+3/2, −*y*+1, *z*+1/2; (iii) *x*+1/2, −*y*+3/2, −*z*+1; (iv) *x*−1/2, −*y*+3/2, −*z*+1; (v) −*x*, *y*+1/2, −*z*+3/2; (vi) *x*−1, *y*, *z*.

### Ring parameters (Å,°)^a^

**Table:**

Ring No.^b^	Source	Q	θ	φ	Conformation
1	here	0.518 (2)	0.0 (2)	120 (11)	Chair
2	here	0.554 (2)	173.2 (2)	159.7 (18)	Chair
2	PUVCIS	0.505 (3)	173.9 (3)	237 (3)	Chair
2	ZENSAM	0.504 (5)	173.5 (6)	174 (5)	Chair
2	GODXEC	0.755 (3)	89.8 (2)	359.6 (3)	Boat
3	here	0.460 (2)	56.7 (2)	59.1 (3)	Envelope
3	PUVCIS	0.478 (3)	54.7 (4)	55.7 (4)	Envelope
3	ZENSAM	0.481 (4)	54.4 (5)	256.6 (6)	Envelope
3	GODXEC	0.538 (3)	86.9 (3)	0.5 (4)	Boat
4	here	na	na	na	(Planar)
4	PUVCIS	0.090 (3)	70.9 (19)	149.6 (19)	Flat-Screw-boat
4	ZENSAM	0.093 (5)	103 (3)	324 (3)	Flat-Twist-boat

a. For puckering parameters, see: Cremer & Pople (1975). b. Ring definitions given in text

### Angles between ring planes (°)^a^

**Table:**

Source	Planes 1,2	Planes 2,3	Planes 1,3
here	63.83 (9)	13.74 (10)	72.85 (10)
PUVCIS	70.52 (13)	16.62 (13)	85.44 (13)
ZENSAM	70.3 (2)	17.0 (2)	85.7 (2)
GODXEC	47.72 (17)	29.33 (15)	76.55 (17)

a. Ring definitions given in text
